# Dose–response relationship between alcohol consumption and workplace absenteeism in Australia

**DOI:** 10.1111/dar.13726

**Published:** 2023-07-30

**Authors:** Melvin Barrientos Marzan, Sarah Callinan, Michael Livingston, Heng Jiang

**Affiliations:** ^1^ Centre for Alcohol Policy Research La Trobe University Melbourne Australia; ^2^ Department of Obstetrics and Gynaecology, Melbourne Medical School University of Melbourne Melbourne Australia; ^3^ Reproductive Epidemiology Group Murdoch Children's Research Institute Melbourne Australia; ^4^ National Drug Research Institute Curtin University Perth Australia; ^5^ Department of Clinical Neurosciences Karolinska Institutet Stockholm Sweden; ^6^ Centre for Health Equity, Melbourne School of Population and Global Health University of Melbourne Melbourne Australia; ^7^ School of Psychology and Public Health La Trobe University Melbourne Australia

**Keywords:** absenteeism, alcohol consumption, Australia, employee, workplace

## Abstract

**Introduction:**

Workplace absenteeism is a burden in Australia. The estimated productivity losses due to alcohol were around $4.0 billion in 2017, with absenteeism driving 90% of these costs. We aim to determine the dose–response relationship between average daily alcohol consumption and heavy episodic drinking (HED) frequency and workplace absenteeism amongst Australian workers.

**Methods:**

We used the 2019 National Drug Strategy Household Survey of Australian employed workers aged ≥20 years to 69 years old. Respondents' average daily alcohol consumption was categorised into four: abstainers, light to moderate (1–20 g of alcohol/day), risky (>20–40 g of alcohol/day) and high‐risk (>40 g of alcohol/day). HED was classified into four frequency measures (never, less than monthly, monthly, weekly). The outcome variables came from dichotomised measures of: (i) absence due to alcohol consumption; and (ii) broader sickness absence–absence due to illness or injury in the previous 3 months.

**Results:**

Risky (adjusted odds ratio 4.74 [95% CI 2.93–7.64]) and high‐risk drinking (adjusted odds ratio 6.61 [95% CI 4.10–10.68]) were linked to increased odds of alcohol‐related absence. Higher HED frequency was significantly associated with alcohol‐related and broader sickness absenteeism. No significant associations exist between regular alcohol consumption and broader sickness absence in fully adjusted models.

**Discussion and Conclusions:**

Findings suggest that only HED is linked to broader sickness absence. However, there is a strong dose–response association between alcohol consumption and alcohol‐related absences for both consumption measures amongst Australian workers. Population‐level policies that reduce alcohol consumption to moderate level and less frequent HED might address workplace absenteeism.

## 1 INTRODUCTION

Work absenteeism is a sizable issue in Australia. The estimated productivity losses due to alcohol were around $4.0 billion (estimated range $1.4 billion—$6.6 billion) in 2017, with absenteeism driving 90% of these costs [1, 2]. Several observational or cohort studies link alcohol consumption and workplace absences [3, 4, 5, 6]. Our dose–response meta‐analysis in 2020 found that, based on the findings of 21 observational studies, risky, high‐risk regular average daily drinking and heavy episodic drinking (HED) are predictive of sickness absence [7]. There are various causal pathways where drinking patterns (HED) and average daily alcohol consumption lead to absenteeism [8, 9, 10, 11, 12, 13, 14]. For example, chronic drinking is associated with more long‐term alcohol‐related morbidities and ill‐health, requiring sick leave [15]. In comparison, HED causes acute effects or injuries from drinking, with hangovers likely to lead to short‐term workplace absences [16, 17, 18, 19, 20]. Earlier studies also noted a higher risk of sickness absence amongst abstainers than moderate drinkers [7, 10]. However, it is often explained as related to confounding ensuing from a ‘sick quitter bias’ (quit drinking due to health reasons) or ‘health‐selection bias’ (never drink or drink avoidance due to health reasons) [13, 21, 22].

The links between drinking and absenteeism are likely to vary by sociodemographic factors, although this has not been widely researched. Some studies have noted sociodemographic and socioeconomic nuances regarding sickness absence [22, 23, 24]. However, differences in absence rates across sub‐groups are not fully explained by alcohol consumption measures, suggesting that alcohol‐related social harms do not simply result from harmful drinking [25]. Hence, it is important to assess how various sociodemographic and socioeconomic indicators interact with alcohol consumption to cause sickness absence to identify groups at disproportionate risk of absenteeism. While our meta‐analysis showed no significant difference in the risk of sickness absence between males and females [7], some studies showed that sex moderates the relationship [24]. Some studies also highlight varying risk levels of sickness absence by age group, with older people having a disproportionately higher risk than younger groups. The excess in older people seems to be related to chronic diseases, which becomes a significant predictor of sickness absence amongst older employees [26]. Marital status is another factor related to sickness absence. Some studies emphasise that married people have better mental health than single or divorced individuals, thus committing lesser work absences [23]. Despite these links with absenteeism generally, there has been little research into how age and marital status moderate the relationship between alcohol and absenteeism.

Several studies found that lower socioeconomic status (SES) groups experience more alcohol‐related harms than expected based on their consumption levels [25, 27, 28, 29]. The distribution of sickness absence tends to be skewed towards groups with lower SES [22, 30] independent of their alcohol consumption, but there has been little work in Australia on this. Research has shown that no single indicator of SES can robustly assess SES across the life course [31]. Thus, we assess the moderating role of SES on the alcohol‐sickness absence relationship using several socioeconomic measures, such as area‐based measures of disadvantage or rurality and individual‐based measures, such as household income, occupation and highest educational attainment.

In Australia, Roche [32] and Pidd [8] analysed the relationship between drinking and absenteeism 15 years ago using cross‐sectional survey data from 2001 and 2004. They found that long‐term (on average, drink over 14 standard drinks per week in the last 12 months) and short‐term (drink five or more standard drinks in a single drinking occasion in the last 12 months) risky drinkers were more likely to be absent from work than moderate and light drinkers. However, trends in alcohol consumption in Australia have changed in the last two decades. For example, young people drink less, while older people's risky drinking rates have remained steady [33]. Alongside this, more females are in the labour force than 20 years ago, and alcohol consumption in middle‐aged females has increased in Australia [33]. There have also been several work‐related policy changes implemented; for example, in 2006, the Australian Council on Trade Unions endorsed the Alcohol and Other Drugs on the Workplace Policy [34]. The policy provides a framework for employers and workers to follow when dealing with issues relating to alcohol consumption and other drugs and to meet their obligations under the relevant occupational health and safety legislation. These changes mean that the links between alcohol consumption and workplace absence identified in earlier studies must be updated to best inform current policy to control alcohol‐related harm in the workplace. More recently, McEntee et al. used the Australian National Drug Strategy Household Survey (NDSHS) 2019 data to extrapolate alcohol‐related work absence amongst Australian workers [1]. The generated prevalence of workplace absences was used to measure the costs of workplace harms caused by alcohol consumption in Australia [1]. However, they only used three categories (i.e., abstainers, alcohol use within safe levels, exceeds safe levels) to measure alcohol use without further disaggregating the alcohol drinking status into average daily alcohol consumption and HED and only included alcohol‐related absences [1]. Our study extended this recent work by providing descriptive and subgroup analyses of alcohol consumption and alcohol‐related and broader sickness work absenteeism measures.

This study examines the relationship between alcohol consumption and alcohol‐related absence and illness or injury‐related sickness absence in Australia using cross‐sectional survey data from 2019. To further flesh out the relationship between various drinking patterns and absences, the relationships between average daily alcohol consumption, frequencies of HED and alcohol‐related absences and key sociodemographic groups will be examined.

## 2 METHODS

### 2.1 Participants and data

Data came from the 2019 NDSHS. The NDSHS is conducted every 3 years and collects data on alcohol and other drug use, attitudes and behaviours, and outcomes relating to substance use [35]. The response rate for the 2019 survey was 49% [33]. A detailed description of the multi‐stage stratified sampling and further details about the study's methodology can be found elsewhere [33].

Of the 22,015 respondents, we first excluded the respondents who were ≥70 years old (*n* = 3934). We then excluded records of respondents less than 20 years old (*n* = 1014) because most were still doing a college level course. We excluded 5836 records of those who were unemployed, retired, students and part‐time workers and 39 records with missing employment data. Out of the remaining 11,394 employed respondents, 1630 were randomly assigned a trial format of the standard alcohol items, whose alcohol data were not comparable with the remaining sample [33] and were thus excluded. There are 9563 records included in our analysis, 1455 (15.1%) have a missing response to alcohol‐related workplace absence questions, and 2511 (26.3%) have a missing response to illness‐injury absences.

### 2.2 Measures

Survey respondents were asked about their alcohol consumption for the previous 12 months, with respondents having consumed any alcohol in the past 12 months classified as current drinkers. The graduated frequency method was used to describe how often (every day, 5–6 days a week, 3–4 days a week, 1–2 days a week, 2–3 days a month, about 1 day a month, less often or never) drinkers had consumed certain amounts of alcohol (in standard drinks of 10 g pure alcohol) per drinking day (20 or more, 11–19, 7–10, 5–6, 3–4, 1–2, less than 1, or none) [33]. The respondents' total annual alcohol consumption was estimated by multiplying the mid‐point of every consumption volume category (e.g., for the 11–19 drinks category, a volume of 15 is used) with the mid‐point of each frequency category (e.g., for 5–6 days per week, a frequency of 5.5*52 = 286 is used) [36]. If the respondents provided more than 365 drinking episodes in the previous year, the maximum drinking episodes were restricted to 365 annually (see [36] for more details). Based on this estimated annual volume, we classified respondents into four groups based on average daily alcohol consumption: abstainers, light to moderate (1–20 g of alcohol/day), risky (20.1–40 g of alcohol/day) and high‐risk (>40 g of alcohol/day) drinking.

Another drinking measure was created based on the number of drinking occasions of five standard drinks following over the past year to derive the HED. HED was defined as drinking more than four‐standard drinks on one occasion [37]. We categorised it into 4 frequency measures (never, less than monthly, monthly, at least weekly). We used these two drinking measures (daily alcohol consumption and HED) across all analyses.

Our outcome measures were dichotomised variables of alcohol‐related and broader sickness absences. Respondents were asked the questions.

*(a) ‘In the last 3 months, how many days of work, school, TAFE or university did you miss because of your use of alcohol?’*

*(b) ‘In the last 3 months, how many days of work, school, TAFE or university did you miss because of any illness/injury?’*

TAFE is an abbreviation for Technical and Further Education institutes, which provides technical and vocational education in Australia. We dichotomised these outcomes into ‘yes’ and ‘no’ since we are only interested to know the prevalence of workplace absenteeism without regard to the number of days missed by employed respondents ensuring compatibility with the previous Australian studies on alcohol consumption and workplace absences.

The relevant Australian and New Zealand Standard Industrial Classification codes were used to determine respondents' occupational groups [38]. We used 7 occupational groups: managers, professionals, trade workers, community service workers, clerical, machinery operators/drivers and labourers. Other sociodemographic covariates were based on the respondent's age, sex, marital status, country of birth, household income and the rurality of their postcode. We used the Index of Relative Socioeconomic Advantage and Disadvantage as one of the measures of Socioeconomic Indexes for Areas [39], based on the respondent's reported postcode. It was coded into quintiles, with 1 as the most disadvantaged and 5 as the least disadvantaged. The Index of Relative Socioeconomic Advantage and Disadvantage is a population‐level indicator of local area disadvantage based on access to social and material resources (Australian Bureau of Statistics) [39].

Daily smoking and pre‐existing health conditions were measured via self‐report. Self‐reported pre‐existing health conditions were diagnosis of insulin‐dependent/non‐dependent diabetes, heart diseases, hypertension, anaemia, asthma, mental disorders, other illnesses or cancers.

### 2.3 Statistical analysis

All analyses were done in Stata version 17 using the ‘SVY’ commands to adjust for the complex survey design and to incorporate survey weights [40]. We executed multiple imputations by chained equation to account for missing data in our dataset which created 6 datasets (full details of multiple imputation included in Supporting Information). The association between average‐daily alcohol consumption (long‐term risk), HED (short‐term risk) and workplace absence outcomes were examined using bivariable and multivariable logistic regression. Covariates included in the model were based on a‐priori from studies on alcohol consumption and workplace absences [6, 8, 10, 11, 32]. We also performed sensitivity analysis by testing the main effects of all our covariates against the model with the covariates and interaction terms to alcohol consumption measures (average daily alcohol consumption) and frequencies of HED. We tested if the main effects of covariates significantly change when the interaction with alcohol consumption measures were added in the model.

We used the post‐estimation command ‘margins’ to estimate the dose–response relationship of average daily alcohol consumption levels by subgroups, mean frequencies of HED, and both measures of absenteeism. We included both the main effects and interactions of all covariates in our dose–response subgroup analyses. The interaction effect was both checked with visual inspections and statistical test. When examining the moderating effect of sociodemographic factors, Wald tests were used to check the significance of each interaction term together with the full set of covariates used in the dose–response modelling. We compared the models with fitted interaction variables against the model without interaction terms.

## 3 RESULTS

Table 1 shows the proportion of respondents who reported taking broader sickness absences and alcohol‐related absences in the last 3 months. There are an estimated 6,542,204 employees who drink alcohol, of which 2.2% (weighted sample = 143,928) reported alcohol‐related absences in the previous 3 months. Concurrently, 26% of all employed respondents reported taking broader sickness absence of 1 day or more in the last 3 months.

**TABLE 1:** Alcohol‐related and illness or broader sickness absences in the last 3 months by average daily alcohol consumption and heavy episodic drinking.^a^

	Broader sickness absence				Alcohol‐related			
	Sample, *n*	Weighted	Proportion absent for 1 day and more	*p*‐value^d^	Sample, *n*	Weighted	Proportion absent for 1 day and more	*p*‐value^d^
Average daily drinking^b^								
Abstainers	916	987,716	23.0%	0.002	1057	1,137,736	‐	<0.0001
Light to moderate	4932	4,764,824	28.3%		5676	5,470,055	1.1%	
Risky	516	525,257	24.3%		601	604,677	5.3%	
High risk	393	367,519	28.4%		449	426,472	9.0%	
Heavy episodic drinking^c^								
Abstainers	485	488,091	23.2%	<0.0001	557	548,586	‐	<0.0001
Never	2941	2,807,615	24.9%		3342	3,201,249	0.7%	
Less than monthly	1229	1,166,786	31.5%		1438	1,363,876	1.4%	
Monthly but less than weekly	1033	1,111,699	29.8%		1203	1,277,783	2.2%	
Weekly or more	1060	1,065,286	28.1%		1235	1,242,599	7.5%	

^a^ Sample size for broader sickness absence and alcohol‐related sickness absence may not match due to missing responses in the workplace absence question.

^b^ Light to moderate (1–20 g of alcohol/day), risky (20.01–40 g of alcohol/day) and high risk (>40 g of alcohol/day) drinkers.

^c^ The Australian National Health and Medical Research Council defines heavy episodic drinking as consuming more than four standard drinks on a single occasion for healthy men and women. Respondents' heavy episodic drinking was classified based on frequency in the past 12 months into never (0), less than monthly (1–11 occasions), monthly but less than weekly (12–51), weekly or more (52+).

^d^ Chi‐square test.

Table 2 shows the results of the multivariable logistic regression predicting the odds of alcohol‐related absences. Compared to light or moderate drinkers, risky (adjusted odds ratio; aOR 4.74 [95% confidence interval; CI 2.93–7.64]) and high‐risk drinkers (aOR 6.61 [95% CI 4.10–10.68]) had increased odds of alcohol‐related absences. Compared to non‐HED—participating in HED monthly (aOR 3.10 [95% CI 1.80–5.34]), and weekly or more (aOR 9.46 [95% CI 5.99–14.93]) HED were both positively associated with increased odds of alcohol‐related absences.

**TABLE 2:** Unadjusted and adjusted odds ratios (OR) for alcohol‐related and broader sickness absences in the last 3 months by alcohol‐consumption categories (with multiple imputations).

	Broader sickness absenteeism								Alcohol‐related absenteeism							
	Unadjusted				Adjusted^a^				Unadjusted				Adjusted^a^			
	OR	95% CI		*p*	OR	95% CI		*p*	OR	95% CI		*p*	OR	95% CI		*p*
Average daily drinking^b^																
Abstainers	0.75	0.63	0.88	0.001	0.76	0.64	0.92	0.005	–				–			
Light to moderate	Ref				Ref				Ref				Ref			
Risky	0.78	0.63	0.96	0.018	0.81	0.66	1.01	0.065	4.87	3.11	7.62	<0.001	4.74	2.93	7.64	<0.001
High risk	0.83	0.65	1.07	0.143	0.90	0.71	1.14	0.396	7.37	4.75	11.44	<0.001	6.61	4.10	10.68	<0.001
Heavy episodic drinking^c^																
Abstainers	0.94	0.71	1.25	0.671	0.95	0.71	1.27	0.737	–				–			
Never	Ref				Ref				Ref				Ref			
Less than monthly	1.37	1.19	1.57	<0.001	1.27	1.10	1.47	<0.001	1.92	0.98	3.73	0.056	1.70	0.86	3.38	0.125
Monthly but less than weekly	1.37	1.16	1.63	<0.001	1.25	1.04	1.50	0.020	3.73	2.17	6.40	<0.001	3.10	1.80	5.34	<0.001
Weekly or more	1.04	0.88	1.23	0.679	1.03	0.86	1.22	0.763	11.17	7.13	17.50	<0.001	9.46	5.99	14.93	<0.001

Abbreviation: CI, confidence interval.

^a^ Adjusted for sex, age group, household income, occupation, highest qualification, Socio‐Economic Indexes for Areas, marital status, daily smoking.

^b^ Light to moderate (1–20 g of alcohol/day), risky (20.01–40 g of alcohol/day) and high risk (>40 g of alcohol/day) drinkers.

^c^ The Australian National Health and Medical Research Council defines heavy episodic drinking as consuming more than four standard drinks on a single occasion for healthy men and women [33]. Respondents' heavy episodic drinking was classified based on frequency in the past 12 months into never (0), less than monthly (1–11 occasions), monthly but less than weekly (12–51), weekly or more (52+).

Turning to broader sickness absences. We found that abstaining (aOR 0.76 [95% CI 0.64–0.92]) is associated with lesser odds of broader sickness absence compared to light to moderate drinking in multivariable models. In terms of HED frequencies, we found that increased participation in HED, such as HED less than monthly (aOR 1.27 [95% CI 1.10–1.47]) and monthly (aOR 1.25 [95% CI 1.04–1.50]) are positively associated with higher odds of broader sickness absence.

In the sensitivity analyses we tested the main effects of all our covariates against the model with both the covariates and interaction terms to alcohol consumption measures (average daily alcohol consumption) and frequencies of HED (please see Table S5, Supporting Information). While there are no meaningful changes in the main effects, the interaction effects of some covariates with significant *p*‐values were attenuated when including the interaction effects. These can be seen in marital status, smoking, age group and presence of comorbidities.

### 3.1 Dose–response subgroup analyses

**FIGURE 1:**
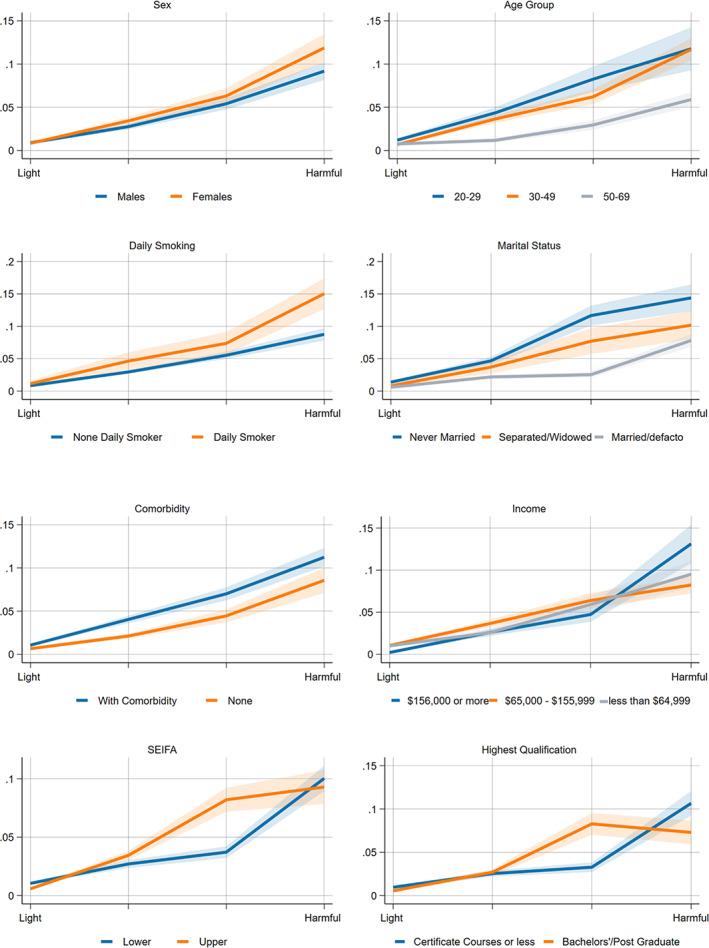
Adjusted (interaction with sex, age group, marital status, smoking status) models for the effect modification of average daily alcohol consumption on self‐reported alcohol‐related absences*. *Visual inspection and significance testing with Wald‐test. The *x*‐axis represents the average daily alcohol consumption such as light to moderate, risky, high‐risk or harmful. Income refers to household income; in terms of Socio‐Economic Indexes for Areas (SEIFA); upper SEIFA means more advantaged socioeconomically advantaged and the lower SEIFA means less socioeconomically advantaged. Wald Test: sex (*p* < 0.001), age group (*p* < 0.001), income (*p* < 0.001), daily smoking (*p* < 0.001), marital status (*p* < 0.001), comorbidity (*p* < 0.001), SEIFA (*p* < 0.001), highest qualification (*p* < 0.001).

**FIGURE 2:**
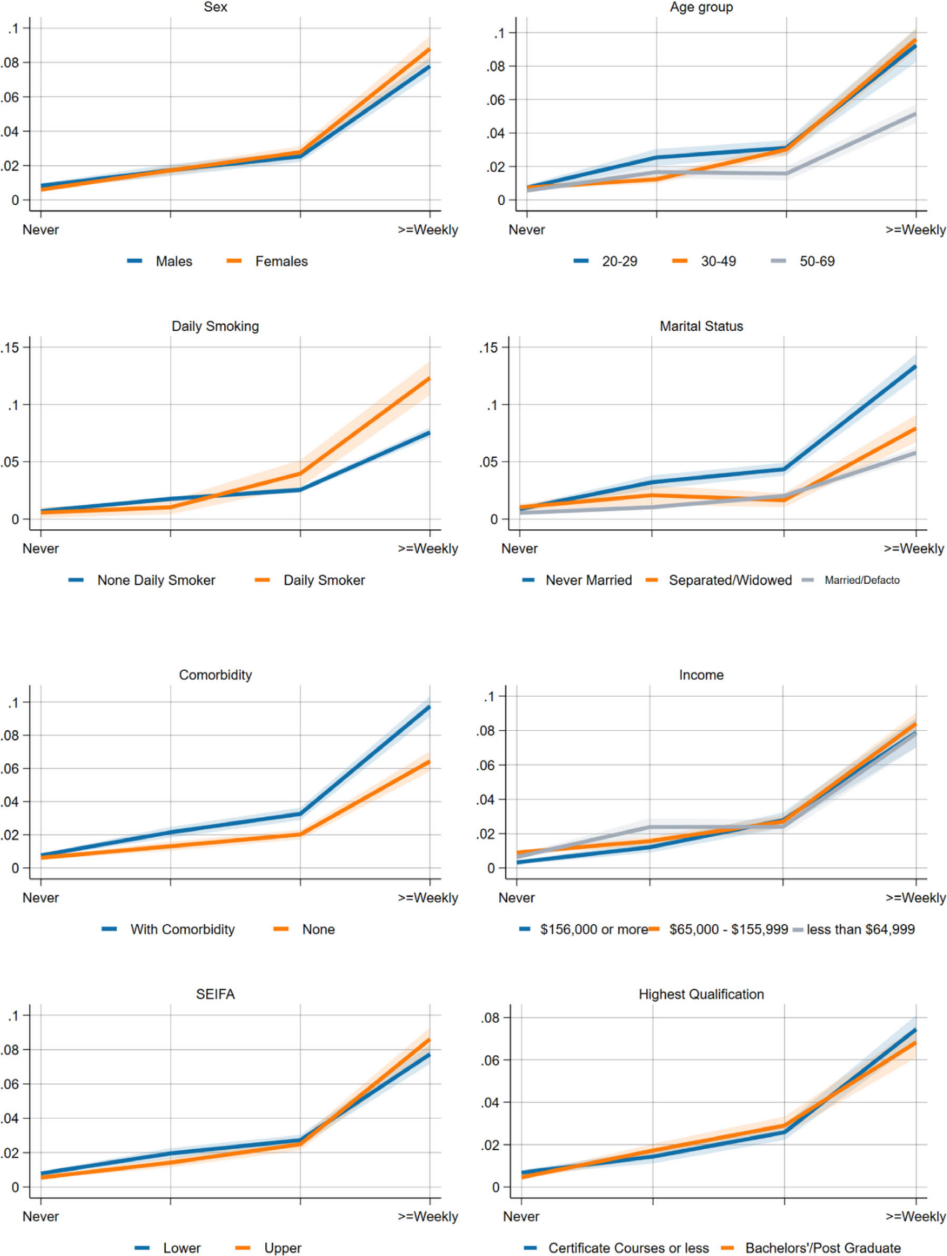
Adjusted (highest education, household income, SEIFA and existing comorbidity) models for the effect modification of heavy episodic drinking on self‐reported alcohol‐related absences. *Visual inspection and significance testing with Wald‐test. The x‐axis represents the average daily alcohol consumption such as light to moderate, risky, high‐risk or harmful. Income refers to household income; in terms of Socio‐Economic Indexes for Areas (SEIFA); upper SEIFA means more advantaged socioeconomically advantaged, and the lower SEIFA means less socioeconomically advantaged. Wald Test: sex (*p* < 0.001), age group (*p* < 0.001), income (*p* < 0.001), daily smoking (*p* < 0.001), marital status (*p* < 0.001), comorbidity (*p* < 0.001), SEIFA (*p* < 0.001), highest qualification (*p* < 0.001).

Figures 1 and 2 show the interaction effects for both measures of drinking and key sociodemographic and socioeconomic measures (the interaction terms for broader sickness absences are in Figures S2 and S3, Supporting Information). The Wald‐tests performed on each sociodemographic variable for alcohol‐related absenteeism demonstrated that all tested covariates have positively significant interactions with the average daily alcohol consumption and annual frequencies of HED in terms of alcohol‐related absences at (*p* < 0.001).

While there are equal probabilities of alcohol‐related absenteeism at low to moderate levels, highly educated, higher income and more advantaged respondents have a greater probability of alcohol‐related absenteeism, particularly at a higher level of alcohol consumption. Both sexes have equal dose–response probability at light to moderate consumption levels. However, females have a higher probability (*p* < 0.001) with increasing alcohol consumption, particularly at risky and high‐risk levels. Regarding age, respondents within the 20–49 age group have a disproportionately higher probability of taking alcohol‐related absences than those 50–69 years old. Meanwhile, daily smokers and those with existing comorbidities tend to have higher probabilities of reporting alcohol‐related absences than those who do not regularly smoke or those without pre‐existing conditions.

For HED frequencies, we see no meaningful effect modification of sex on alcohol‐related absences on visual inspection, although the Wald test noted a significant difference between males and females. The differences amongst the age group, Socioeconomic Indexes for Areas, income and daily smoking were only noticeable at the higher frequencies of HED (*p* < 0.001). Those who were never married, have comorbidities and have higher educational attainment have a disproportionately higher risk of alcohol‐related absences than other comparative groups.

## 4 DISCUSSION

This paper estimated the association between alcohol consumption and workplace absences using a representative Australian cross‐sectional survey. We expanded the previous analyses [8, 32] by examining interaction effects between sociodemographic and health covariates, alcohol consumption and workplace absences. Thus, we provided information on what subgroups were disproportionately impacted by alcohol consumption in relation to social harms. Based on our calculations, alcohol consumption accounts for around 8% of all workplace absences in Australia annually. Our study extended the results of previous Australian studies, which only used the dichotomised drinking measure of abstainers and alcohol consumers [1]. We provided more robust exposure measures of alcohol consumption by using the average daily drinking consumption (drinking volume) and patterns of drinking (HED) – both likely linked to workplace absenteeism in different ways. Furthermore, this study measured two outcomes to understand the association between heavy drinking patterns and daily alcohol consumption and workplace absenteeism.

### 4.1 Alcohol‐related absences

We noted a lower proportion of alcohol‐related absences at 2.2% in 2019 compared to the previous studies that used 2001 (3.5%) and 2004 (3.7%) NDSHS data [8, 32]. This may partially be related to the significant decline in population‐level consumption in Australia over the same period [33]. We also found a clear positive dose–response relationship between average alcohol consumption, frequencies of HED and alcohol‐related absences in 2019, in line with previous Australian studies using 2001 and 2004 data [8, 32]. Our study supported previous work that found drinking beyond the moderate thresholds and at higher frequencies of HED are associated with an increased risk of alcohol‐related absences amongst Australian workers [8, 32].

### 4.2 Broader sickness absences

Overall, we found an ambiguous dose–response relationship between average daily alcohol consumption and broader sickness absences, with abstaining and risky‐level drinking both negatively associated with broader sickness absence. However, it can be more attributed to confounding that may emanate from the ‘prevention paradox’ since most respondents are low‐to‐moderate drinkers [41]. However, we found that increased frequencies in participation in HED are significantly positively associated with broader sickness absence. This is in line with the previous Australian studies [8, 32] that found that increased HED frequencies were positively significantly linked to broader sickness absence. It may relate to earlier findings that participation in HED is associated with acute effects or injuries from drinking, which might likely lead to short‐term workplace absences [16, 17, 18, 19, 20].

### 4.3 Dose–response subgroup analyses

To our knowledge, this is one of the few studies that examined whether the links between alcohol consumption and workplace absenteeism varied across demographic, socioeconomic and health factors using interaction analysis. Our interaction models showed that the risk of alcohol‐related absence for each sex was roughly the same until the moderate level. At that point, women's probability of alcohol‐related absence increased more quickly. This likely reflects the relative sensitivity of females to the effect of alcohol than males [42].

We also found that younger and unmarried respondents had a higher probability of alcohol‐related absences than older and married respondents at the same dose or frequency of drinking. These differences may reflect the higher prevalence of hazardous drinking beyond what we measured in this study amongst younger age groups or higher rates of risky behaviour more generally [43]. How marital status modifies the relationship between alcohol consumption and alcohol‐related absences is unclear; however, an Australian study suggested that lower prevalence of risky behaviours and alcohol‐related harms amongst married people may partly be associated with the better mental health status of married subjects when compared against unmarried and separated respondents [44] in addition to parental roles which likely reduce the drinking frequencies of married respondents [45].

On socioeconomic status, we did not see any evidence that lower socioeconomic groups are consistently at a higher risk of alcohol‐related absence for a given level of daily drinking or HED frequency. We found that respondents in the higher socioeconomic position have a higher probability of alcohol‐related sickness absence until high‐risk levels of average daily alcohol consumption. Our findings deviate from previous studies that found the association between alcohol use and sickness absence differed in various socioeconomic groups [23, 30, 46]. Though, our study may not be directly comparable since the previous studies did not use interaction analysis to account for the effect modification of SES on alcohol consumption and sickness absence. This finding can also be explained partly by some disparity in working arrangements as people in higher socioeconomic positions generally have better working arrangements, such as flexible working hours and more sick leave [22]. However, a more robust study including a larger sample size would be further needed to confirm the association of working arrangements to sickness absence. Furthermore, previous studies noted that the socioeconomic gradients on the impact of alcohol consumption on overall sickness absence are primarily present in long‐term absences and may not be reflected in short‐term absences [30], which may also explain the lack of a strong interaction in our analyses. Furthermore, previous studies noted that the socioeconomic gradients on the impact of alcohol consumption on overall sickness absence are primarily present in long‐term absences and may not be reflected in short‐term absences [30], which may also explain the lack of a strong interaction in our analyses. Furthermore, previous studies noted that the socioeconomic gradients on the impact of alcohol consumption on overall sickness absence are primarily present in long‐term absences and may not be reflected in short‐term absences [30], which could not be extrapolated from the present design of the NDSHS.

### 4.4 Limitations

There are some caveats to our study. First, both the exposure (alcohol consumption) and outcomes (illness/injury and alcohol‐related absenteeism) were measured simultaneously at a one‐time point; thus, we cannot derive a causal relationship from the data. All measures are self‐reported and likely affected by the well‐known problems of self‐report data [47]. For example, there might be an underestimation on the alcohol consumption amongst the respondents as documented earlier [48]. This underestimation might result to lower odds ratio of the higher threshold for average daily alcohol consumption and more frequencies of HED. The workplace absence question also entails the respondents to classify whether they have committed alcohol‐related absences or illness or injury‐related. The question might have caused some misclassification and ambiguity amongst the respondents. We have inadequate information to check the accuracy of the response to the outcomes' questions. However, the incremental dose–response association between average daily alcohol consumption and frequencies of HED and alcohol‐related sickness absence might reflect an acceptable degree of attribution to alcohol on alcohol‐related absences the respondent's committed. We used the alcohol consumption variable based on the self‐reported estimated daily alcohol consumption rather than the Alcohol Use Disorders Identification Test questionnaire variable. This ensures consistency with our previous study on alcohol consumption and sickness absence [7] and similar previous studies in Australia [8, 32, 49]. Moreover, we dichotomised the outcomes of workplace absence measures which may conceal the attribution of alcohol consumption to both short‐ and long‐term workplace absences. These are done to be consistent with the previous Australian studies on alcohol consumption and workplace absenteeism [8, 32] and ensure comparison of our results. Lastly, the dose–response modelling categorised the underlying continuous measures of daily alcohol consumption and HED, which may cause inadequate power and misclassification errors [50]. Future research should use linked datasets or cohort studies that rely on recorded sickness absence from a registry rather than self‐report. This will help better understand the relationship between alcohol consumption and sickness absence amongst the Australian population.

## 5 CONCLUSION

We found a positive dose–response relationship between average daily alcohol consumption and alcohol‐related absences. Our study also demonstrated a significant association between increased frequencies of HED to the broader sickness absence. The relationship between alcohol consumption and alcohol‐related sickness absence varies by sociodemographic factors such as sex, age and marital status, daily smoking and existing illnesses. Moreover, the burden of alcohol‐related absences was disproportionately highest amongst most frequent heavy episodic drinkers and risky daily drinkers. Thus, population‐level policies like increasing alcohol prices that reduce alcohol consumption levels to light and moderate and less frequent HED should effectively address alcohol‐related work absences and broader sickness absences. Controlling alcohol outlet density and enforcing alcohol trading hours may also help to address workplace absenteeism amongst Australian workers.

## AUTHOR CONTRIBUTIONS

Each author certifies that their contribution to this work meets the standards of the International Committee of Medical Journal Editors.. MM: Writing of the primary manuscript Data analysis plan, data cleaning and analysis, creation of figures and tables, data interpretation. SC: Data interpretation, reviewing and editing the draft manuscript for intellectual content, approval of the final submitted manuscript. ML: Data interpretation, reviewing and editing the draft manuscript for intellectual content, approval of the final submitted manuscript. HJ: Overall supervision of the project, reviewing and editing the draft manuscript for intellectual content, approval of the final submitted manuscript.

## CONFLICT OF INTEREST STATEMENT

The authors declared they have no conflicts of interest.

## Supporting information


**Data S1:** Supporting information.

**Data S2:** Supporting information.
