# Supplementary material for: Dose–response relationship between alcohol consumption and workplace absenteeism in Australia
Source: Drug Alcohol Rev. 2023 Jul 30;42(7):1773–84. doi: 10.1111/dar.13726 (PMC10947312; doi:10.1111/dar.13726)
Supplement: Supplementary file 1 — Data S1: Supporting information. [file DAR-42-1773-s002.docx]

**Figure S1.** Histogram of the number of alcohol-related and broader sickness absence

**Table S1.** Alcohol-related and broader sickness absences in the last 3 months by age and sex

|  | All workers | | |  |  |  | Men workers | | Women workers | |
| --- | --- | --- | --- | --- | --- | --- | --- | --- | --- | --- |
| Age, years | Sample, n | Proportion absent for 1 day and more, % | Bivariable analysis (Odds) | | | P-value | Sample, n | Proportion absent for 1 day and more, % | n | Proportion absent for 1 day and more, % |
|  |  |  | OR | lb | ub |  |  |  |  |  |
| **Broader sickness absence*** | | | | | | | | | | |
| 20-29 | 1036 | 35.1 | ref |  |  |  | 472 | 32.6 | 564 | 37.2 |
| 30-39 | **1792** | **32.5** | **0.83** | **0.79** | **0.87** | **<0.001** | 835 | 31.6 | 957 | 33.3 |
| 40-49 | **1713** | **25.7** | **0.61** | **0.58** | **0.64** | **<0.001** | 785 | 22.6 | 928 | 28.5 |
| 50-59 | **1599** | **21.6** | **0.49** | **0.47** | **0.52** | **<0.001** | 768 | 19.4 | 831 | 23.6 |
| 60+ | **912** | **17.0** | **0.37** | **0.35** | **0.39** | **<0.001** | 480 | 15.0 | 432 | 19.2 |
| **Alcohol-related absence**** | | | | | | | | | | |
| 20-29 | 1228 | 3.3 | ref |  |  |  | 560 | 3.8 | 20 | 3.0 |
| 30-39 | 2071 | 2.2 | **0.51** | **0.44** | **0.59** | **<0.001** | 965 | 2.9 | 17 | 1.5 |
| 40-49 | **1957** | **2.0** | **0.52** | **0.44** | **0.61** | **<0.001** | 909 | 2.4 | 18 | 1.7 |
| 50-59 | **1829** | **1.4** | **0.34** | **0.29** | **0.41** | **<0.001** | 860 | 1.4 | 14 | 1.4 |
| 60+ | **1033** | **0.7** | **0.17** | **0.13** | **0.22** | **<0.001** | 545 | 0.7 | 3 | 0.6 |

*Chi-square p-value <0.0001. **Chi-square p-value <0.0001.

OR, odds ratio.

**Table S2.** Alcohol-related and illness or injury absences in the last 3 months by age, occupation, educational attainment, household income and marital status, sex and SEIFA

|  | Illness/Injury absenteeism | | | | |  | Alcohol-related absenteeism | | | | |  |
| --- | --- | --- | --- | --- | --- | --- | --- | --- | --- | --- | --- | --- |
|  | Sample, n | Proportion absent for ≥1 day | Bivariate analysis (Odds) | | | P-value | Sample, n | Proportion absent for ≥1 day | Adjusted (Odds) | | | P-value |
|  |  |  | OR | lb | ub |  |  |  | OR | lb | ub |  |
| **Occupation** |  |  |  |  |  |  |  |  |  |  |  |  |
| Managers | **1133** | **23.7%** | **0.70** | **0.67** | **0.73** | 0.00 | 1283 | 2.0% | 0.81 | 0.71 | 0.93 | 0.00 |
| Professionals | **2430** | **34.2%** | ref | | | | 2853 | 2.0% | ref | | | |
| Technicians/trade workers | **958** | **23.1%** | **0.69** | **0.66** | **0.72** | 0.00 | 1116 | 2.8% | 1.21 | 1.05 | 1.41 | 0.01 |
| Community/personal service | 877 | 28.1% | 0.83 | 0.79 | 0.86 | 0.00 | 1034 | 2.8% | 1.11 | 0.96 | 1.28 | 0.15 |
| Clerical /administrative | 1570 | 28.7% | 0.85 | 0.82 | 0.88 | 0.00 | 1809 | 3.0% | 1.18 | 1.05 | 1.33 | 0.01 |
| Machinery operators/drivers | **392** | **18.1%** | **0.57** | **0.53** | **0.61** | 0.00 | 454 | 2.4% | 1.10 | 0.90 | 1.33 | 0.35 |
| Labourers | **506** | **26.0%** | **0.70** | **0.66** | **0.75** | 0.00 | 556 | 2.2% | 1.35 | 1.15 | 1.57 | 0.00 |
| **Educational attainment** | | | | | | | | | | | |  |
| High school or less | 108 | 15.5% | 0.46 | 0.42 | 0.51 | 0.00 | 124 | 1.3% | 0.80 | 0.61 | 1.04 | 0.10 |
| Certificate or diploma | **3124** | **25.8%** | **0.80** | **0.78** | **0.83** | 0.00 | 3626 | 2.5% | 1.23 | 1.11 | 1.35 | 0.00 |
| Bachelor Degree | 1982 | 30.3% | ref | | | | 2289 | 1.6% | ref | | | |
| Postgraduate (Masters/PhD) | 1380 | 34.1% | 1.11 | 1.07 | 1.16 | 0.00 | 1612 | 1.9% | 0.85 | 0.74 | 0.97 | 0.02 |
| **Household income** | | | | | | | | | | | |  |
| $156,000 or more | 2409 | 29.8% | ref | | | | 2765 | 2.4% | ref | | | |
| $65,000 - $155,999 | 3606 | 29.6% | 0.94 | 0.91 | 0.97 | 0.00 | 4230 | 2.5% | 1.03 | 0.92 | 1.14 | 0.62 |
| Less than $64,999 | 1472 | 24.7% | 0.74 | 0.72 | 0.77 | 0.00 | 1687 | 2.8% | 0.99 | 0.89 | 1.12 | 0.93 |
| **Marital status** | | | | | | | | | | | |  |
| Never married | 1714 | 35.3% | ref | | | | 2028 | 5.0% | ref | | | |
| Divorced/separated/widowed | **1001** | **27.4%** | **0.65** | **0.62** | **0.68** | **0.00** | **1163** | **2.0%** | **0.39** | **0.34** | **0.45** | **0.00** |
| Married/defacto | **5592** | **25.3%** | **0.62** | **0.61** | **0.65** | **0.00** | **6416** | **1.5%** | **0.28** | **0.26** | **0.30** | **0.00** |
| **Sex** | | | | | | | | | | | | |
| Men | **3947** | **26.3%** | **0.86** | **0.84** | **0.88** | **0.00** | **4550** | **2.8%** | **1.53** | **1.41** | **1.66** | **0.00** |
| Women | 4374 | 29.5% | ref | | | | 5076 | 1.8% | ref | | | |
| **SEIFA quintile** | | | | | | | | | | | | |
| Lowest (most disadvantaged) | 2842 | 26.9% | 0.94 | 0.91 | 0.97 | 0.00 | 3276 | 2.4% | 1.01 | 0.92 | 1.10 | 0.87 |
| Middle | 3499 | 28.5% | ref | | | | 4063 | 2.6% | ref | | | |
| Highest (least disadvantaged) | **1980** | **27.7%** | 0.97 | 0.93 | 1.00 | 0.07 | 2287 | 2.1% | 0.82 | 0.73 | 0.92 | 0.00 |

OR, odds ratio; SEIFA, Socio-Economic Indexes for Areas.

**Table S3.** Alcohol-related and illness or injury absences in the last 3 months by country of birth, rurality, smoking status and co-morbidity

|  | Illness/Injury absenteeism | | |  |  |  | Alcohol-related absenteeism | | |  |  |  |
| --- | --- | --- | --- | --- | --- | --- | --- | --- | --- | --- | --- | --- |
|  | Sample, n | Proportion absent for ≥1 day | Bivariate analysis (Odds) | | | P-value | Sample, n | Proportion absent for ≥1 day | Bivariate analysis (Odds) | | | P-value |
|  |  |  | OR | lb | ub |  |  |  | OR | lb | ub |  |
| **Country of birth** | | | | | | | | | | | | |
| Australia | 6012 | 29.6% | ref | | | | 6956 | 2.58% | ref | | | |
| Other countries | 1935 | 23.2% | 0.76 | 0.74 | 0.78 | 0.00 | 2241 | 2.07% | 0.71 | 0.65 | 0.79 | 0.00 |
| **Rurality** | | | | | | | | | | | | |
| Major cities | 3450 | 29.5% | ref | | | | 4060 | 2.29% | ref | | | |
| Inner regional | 1019 | 24.9% | 0.81 | 0.78 | 0.83 | 0.00 | 1140 | 1.84% | 0.75 | 0.67 | 0.83 | 0.00 |
| Outer regional/remote | 237 | 27.8% | 0.79 | 0.75 | 0.84 | 0.00 | 265 | 2.00% | 0.53 | 0.41 | 0.68 | 0.00 |
| **Daily smoking status** | | | | | | | | | | | | |
| Not smoking | 8066 | 27.7% | ref | | | | 9299 | 2.20% | ref | | | |
| Smoking | 255 | 29.4% | 1.01 | 0.94 | 1.08 | 0.80 | 327 | 7.58% | 3.15 | 2.78 | 3.57 | 0.00 |
| **Presence of comorbidities** | | | | | | | | | | | | |
| None | 3862 | 31.8% | ref | | | | 4576 | 2.97% | ref | | | |
| With comorbidities | 4419 | 24.5% | 1.38 | 1.34 | 1.42 | 0.00 | 5004 | 1.87% | 1.39 | 1.27 | 1.51 | 0.00 |

OR, odds ratio.

**Table S4.** Adjusted and adjusted with interaction terms odds ratios (OR) for alcohol-related and broader sickness absences in the last 3 months by alcohol-consumption categories (with multiple imputations)

|  | **Broader sickness absenteeism** | | | | | | | | **Alcohol-related absenteeism** | | | | | | | |
| --- | --- | --- | --- | --- | --- | --- | --- | --- | --- | --- | --- | --- | --- | --- | --- | --- |
|  | Adjusted* | | | | With interactions | | | | Adjusted* | | | | With interactions | | | |
|  | OR | 95% CI | | P | OR | 95% CI | | P | OR | 95% CI | | P | OR | 95% CI | | P |
| **Average daily drinking^A^** |  |  |  |  |  |  |  |  |  |  |  |  |  |  |  |  |
| Abstainers | **0.82** | **0.78** | **0.86** | **0.00** | 0.60 | 0.50 | 0.72 | **0.00** | – |  |  |  | – |  |  |  |
| Light to moderate | Ref |  |  |  | Ref |  |  |  | Ref |  |  |  | Ref |  |  |  |
| Risky | **0.83** | **0.78** | **0.89** | **0.00** | **0.62** | **0.48** | **0.79** | **0.00** | **3.55** | **3.06** | **4.12** | **<0.0001** | **9.54** | **5.80** | **15.67** | **<0.0001** |
| High risk | 0.99 | 0.91 | 1.07 | 0.79 | 1.20 | 0.90 | 1.60 | 0.79 | **5.83** | **5.01** | **6.78** | **<0.0001** | **7.76** | **4.47** | **13.48** | **<0.0001** |
| **Heavy episodic drinking^B^** |  |  |  |  |  |  |  |  |  |  |  |  |  |  |  |  |
| Abstainers | **0.97** | **0.91** | **1.04** | **0.44** | 1.14 | 0.84 | 1.56 | 0.39 |  | – |  |  |  | – |  |  |
| Never | Ref |  |  |  | Ref |  |  |  | Ref |  |  |  | Ref |  |  |  |
| Less than monthly | **1.25** | **1.19** | **1.31** | **<0.0001** | **1.35** | **1.13** | **1.61** | **0.00** | **2.01** | **1.60** | **2.52** | **<0.0001** | **4.26** | **2.23** | **8.12** | **<0.0001** |
| Monthly but less than weekly | **1.14** | **1.08** | **1.20** | **<0.0001** | **1.19** | **1.00** | **1.41** | 0.06 | **2.96** | **2.42** | **3.63** | **<0.0001** | **3.19** | **1.61** | **6.29** | **<0.0001** |
| Weekly or more | **1.11** | **1.05** | **1.17** | **<0.0001** | **0.85** | **0.70** | **1.03** | 0.10 | **10.14** | **8.47** | **12.13** | **<0.0001** | **22.90** | **12.96** | **40.45** | **<0.0001** |

* Adjusted for sex, age group, household income, occupation, highest qualification, Socio-Economic Indexes for Areas, marital status, daily smoking, co-morbidity.

** Interaction terms include sex, age group, household income, occupation, highest qualification, Socio-Economic Indexes for Areas, marital status, daily smoking, co-morbidity and occupation.

^A^ Light to moderate (1-20 grams of alcohol/day), risky (20.01-40 grams of alcohol/day) and high risk (>40 grams of alcohol/day) drinkers.

^B^ The Australian National Health and Medical Research Council defines heavy episodic drinking as consuming more than four standard drinks on a single occasion for healthy men and women [34]. Respondents’ heavy episodic drinking was classified based on frequency in the past 12 months into never (0), less than monthly (1-11 occasions), monthly but less than weekly (12-51), weekly or more (52+).

CI, confidence interval.

**Table S5.** Main effects of sociodemographic, health and socioeconomic covariates versus the interaction with alcohol consumption measures in alcohol-related work absences in the last 3 months by alcohol-consumption categories (with multiple imputations)

|  | Alcohol-related sickness absence (average daily alcohol consumption) | | | | | | | | Alcohol-related sickness absence (heavy episodic drinking) | | | | | | | |
| --- | --- | --- | --- | --- | --- | --- | --- | --- | --- | --- | --- | --- | --- | --- | --- | --- |
| Covariates | Adjusted main effects | | | P-value | With interactions | | | P-value | Adjusted main effects | | | P-value | With interactions | | | P-value |
|  | (Odds ratio) | | |  | (Odds ratio) | | |  | (Odds ratio) | | |  | (Odds ratio) | | |  |
|  | OR | lb | ub |  | OR | lb | ub |  | OR | lb | ub |  | OR | lb | ub |  |
| **Occupation** |  |  |  |  |  |  |  |  |  |  |  |  |  |  |  |  |
| Managers | 0.95 | 0.50 | 1.79 | 0.87 | 1.55 | 0.75 | 3.22 | 0.24 | 0.98 | 0.52 | 1.85 | 0.95 | 0.50 | 0.11 | 2.35 | 0.38 |
| Professionals | ref |  |  |  | ref |  |  |  | ref |  |  |  | ref |  |  |  |
| Technicians/trade workers | 0.71 | 0.35 | 1.44 | 0.33 | 1.23 | 0.54 | 2.82 | 0.62 | 0.70 | 0.37 | 1.35 | 0.29 | 0.67 | 0.31 | 1.48 | 0.33 |
| Community/personal service | 0.88 | 0.41 | 1.86 | 0.73 | 1.00 | 0.44 | 2.31 | 0.99 | 0.87 | 0.43 | 1.77 | 0.69 | 0.81 | 0.21 | 3.16 | 0.76 |
| Clerical/administrative | 1.21 | 0.70 | 2.11 | 0.49 | 2.15 | 1.16 | 4.01 | 0.02 | 1.18 | 0.68 | 2.04 | 0.56 | 1.29 | 0.46 | 3.63 | 0.63 |
| Machinery operators/drivers | 0.66 | 0.26 | 1.63 | 0.36 | 0.29 | 0.04 | 2.25 | 0.24 | 0.70 | 0.29 | 1.69 | 0.43 | 0.82 | 0.30 | 2.24 | 0.70 |
| Labourers | 0.56 | 0.25 | 1.29 | 0.17 | 1.12 | 0.40 | 3.15 | 0.83 | 0.57 | 0.25 | 1.31 | 0.19 | 0.52 | 0.20 | 1.41 | 0.20 |
| **Age group, years** |  |  |  |  |  |  |  |  |  |  |  |  |  |  |  |  |
| 20-29 | 1.15 | 0.75 | 1.76 | 0.53 | 1.08 | 0.60 | 1.94 | 0.81 | 1.01 | 0.66 | 1.57 | 0.95 | 1.19 | 0.35 | 4.08 | 0.78 |
| 30-49 | ref |  |  |  | ref |  |  |  | ref |  |  |  | ref |  |  |  |
| 40-49 | 0.51 | 0.33 | 0.78 | 0.00 | 0.53 | 0.29 | 0.96 | 0.04 | 0.61 | 0.39 | 0.95 | 0.03 | 0.65 | 0.26 | 1.61 | 0.35 |
| **Educational attainment** |  |  |  |  |  |  |  |  |  |  |  |  |  |  |  |  |
| Certificate or Diploma or less | ref |  |  |  | ref |  |  |  | ref |  |  |  |  |  |  |  |
| Bachelor Degree and above | 0.97 | 0.58 | 1.63 | 0.91 | 0.78 | 0.40 | 1.53 | 0.46 | 0.98 | 0.61 | 1.58 | 0.94 | 0.55 | 0.23 | 1.31 | 0.18 |
| **Household income** |  |  |  |  |  |  |  |  |  |  |  |  |  |  |  |  |
| $156,000 or more | ref |  |  |  | ref |  |  |  | ref |  |  |  | ref |  |  |  |
| $65,000 - $155,999 | 1.09 | 0.73 | 1.62 | 0.68 | 1.20 | 0.69 | 2.06 | 0.52 | 1.21 | 0.82 | 1.80 | 0.34 | 3.36 | 0.58 | 19.48 | 0.17 |
| less than $64,999 | 0.88 | 0.50 | 1.53 | 0.65 | 0.86 | 0.41 | 1.79 | 0.68 | 1.04 | 0.61 | 1.77 | 0.90 | 2.63 | 0.25 | 27.48 | 0.40 |
| **Marital status** |  |  |  |  |  |  |  |  |  |  |  |  |  |  |  |  |
| Never married | ref |  |  |  | ref |  |  |  | ref |  |  |  | ref |  |  |  |
| Divorced/separated/widowed | 0.53 | 0.30 | 0.94 | 0.03 | 0.68 | 0.31 | 1.49 | 0.34 | 0.54 | 0.30 | 0.99 | 0.05 | 1.04 | 0.28 | 3.83 | 0.95 |
| Married/defacto | 0.39 | 0.26 | 0.57 | 0.00 | 0.46 | 0.27 | 0.78 | 0.00 | 0.43 | 0.28 | 0.64 | 0.00 | 0.62 | 0.20 | 1.89 | 0.40 |
| **Sex** |  |  |  |  |  |  |  |  |  |  |  |  |  |  |  |  |
| Men | ref |  |  |  | ref |  |  |  | ref |  |  |  | ref |  |  |  |
| Women | 0.88 | 0.60 | 1.30 | 0.53 | 0.74 | 0.45 | 1.22 | 0.24 | 0.98 | 0.66 | 1.46 | 0.94 | 0.69 | 0.28 | 1.66 | 0.40 |
| **SEIFA quintile** |  |  |  |  |  |  |  |  |  |  |  |  |  |  |  |  |
| Lowest | 0.81 | 0.55 | 1.18 | 0.27 | 0.86 | 0.49 | 1.50 | 0.59 | 0.82 | 0.55 | 1.20 | 0.31 | 0.65 | 0.26 | 1.63 | 0.36 |
| Middle | ref |  |  |  | ref |  |  |  | ref |  |  |  | ref |  |  |  |
| Highest | 0.75 | 0.48 | 1.17 | 0.20 | 0.77 | 0.42 | 1.40 | 0.39 | 0.74 | 0.47 | 1.17 | 0.19 | 0.38 | 0.11 | 1.35 | 0.13 |
| **Rurality** |  |  |  |  |  |  |  |  |  |  |  |  |  |  |  |  |
| Major cities | ref |  |  |  | ref |  |  |  | ref |  |  |  |  |  |  |  |
| Inner regional | 0.95 | 0.43 | 2.10 | 0.90 | 0.58 | 0.31 | 1.09 | 0.09 | 0.93 | 0.42 | 2.09 | 0.85 | 0.53 | 0.05 | 5.97 | 0.58 |
| Outer regional/remote | 0.82 | 0.26 | 2.56 | 0.72 | 0.78 | 0.28 |  | 0.64 | 0.83 | 0.27 | 2.55 | 0.73 | 0.97 | 0.13 | 7.23 | 0.98 |
| **Daily smoking status** |  |  |  |  |  |  |  |  |  |  |  |  |  |  |  |  |
| Not smoking | ref |  |  |  | ref |  |  |  | ref |  |  |  |  |  |  |  |
| Smoking | 2.38 | 1.42 | 3.99 | 0.00 | 1.75 | 0.69 | 4.43 | 0.24 | 2.25 | 1.34 | 3.77 | 0.00 | 1.46 | 0.19 | 10.98 | 0.72 |
| **Presence of comorbidities** |  |  |  |  |  |  |  |  |  |  |  |  |  |  |  |  |
| None | ref |  |  |  | ref |  |  |  | ref |  |  |  |  |  |  |  |
| With comorbidities | 1.65 | 1.19 | 2.29 | 0.00 | 1.84 | 1.11 | 3.05 | 0.02 | 1.68 | 1.21 | 2.33 | 0.00 | 0.90 | 0.36 | 2.24 | 0.82 |

Adjusted for occupation, educational attainment, household income, marital status, sex, seifa quintile, rurality, daily smoking status, and presence of comorbidities.

SEIFA, Socio-Economic Indexes for Areas.

**Figure S2.** Adjusted (interaction with gender, age group, marital status, highest education, household income, SEIFA, smoking status and existing comorbidity) models for the effect modification of annual average heavy episodic drinking frequencies on self-reported alcohol-related absences


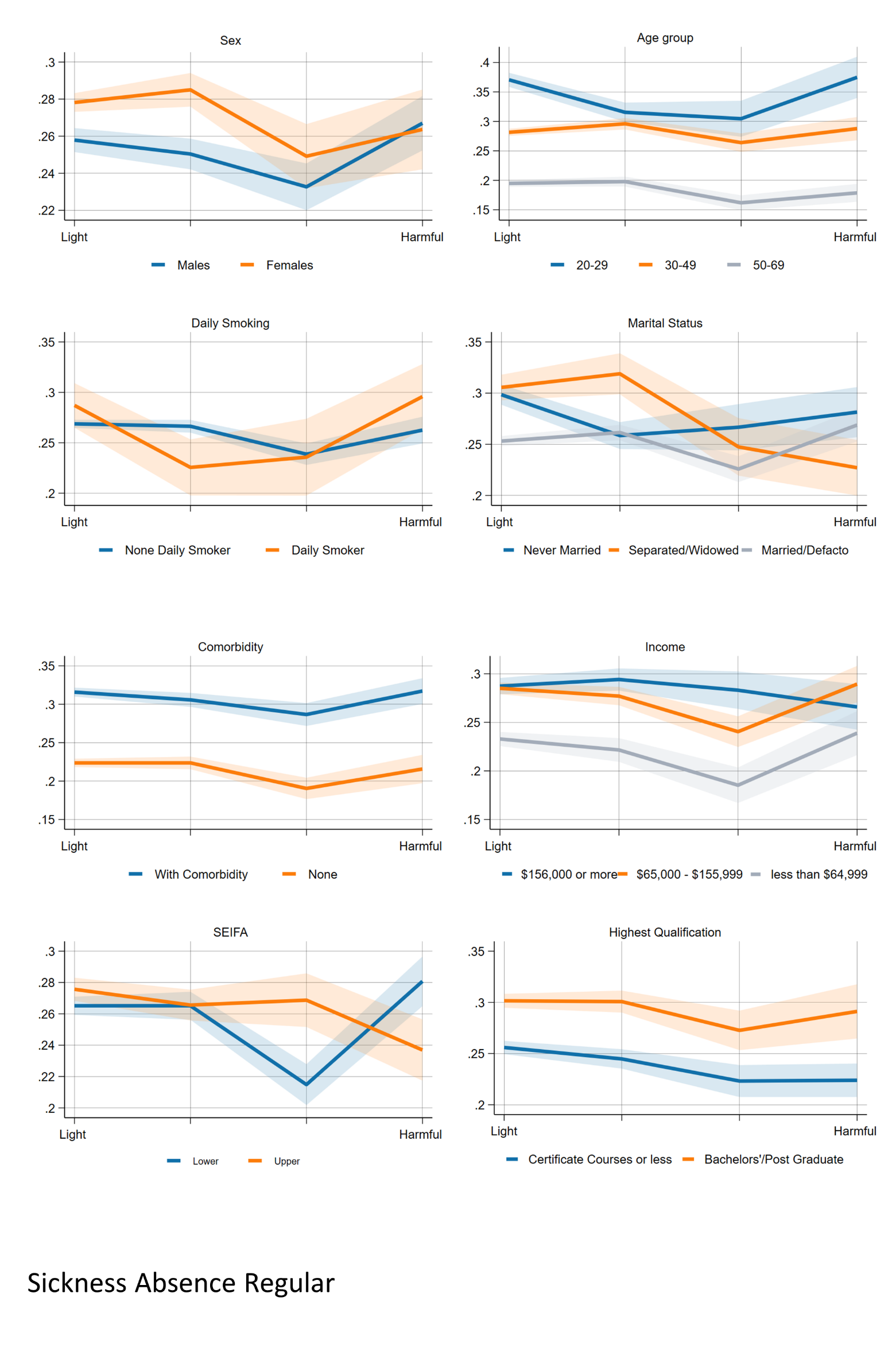


Wald Test: Sex (p=1.55), Age group (p <0.001), Income (p=0.05), Daily smoking (p=0.21), Marital status (p=0.23), Comorbidity (p <0.001), SEIFA (p <0.12), Highest Qualification (p=0.03).

SEIFA, Socio-Economic Indexes for Areas.

**Figure S3.** Adjusted (interaction with sex, age group, marital status, highest education, household income, SEIFA, smoking status and existing comorbidity) models for the effect modification of annual average heavy episodic drinking frequencies on self-reported illness/injury absences


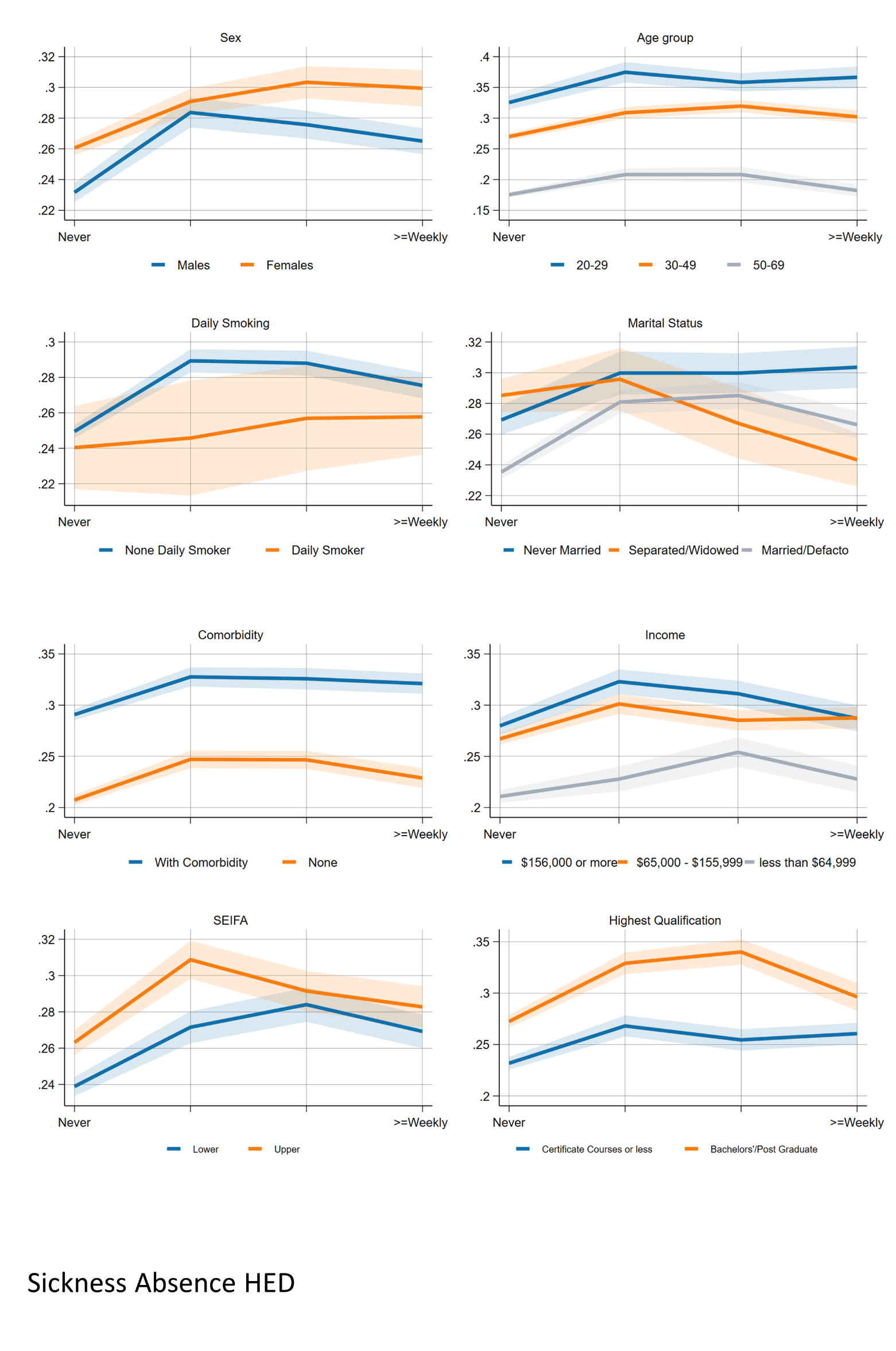
Wald Test: Sex (p=0.03), Age group(p <0.001), Income(p=0.05), Daily smoking (p=0.13), Marital status (p=0.11), Comorbidity (p <0.001), SEIFA (p=0.33), Highest qualification (p=0.08).

SEIFA, Socio-Economic Indexes for Areas.
