# Supplementary material for: Dose–response relationship between alcohol consumption and workplace absenteeism in Australia
Source: Drug Alcohol Rev. 2023 Jul 30;42(7):1773–84. doi: 10.1111/dar.13726 (PMC10947312; doi:10.1111/dar.13726)
Supplement: Supplementary file 2 — Data S2: Supporting information. [file DAR-42-1773-s001.pdf]

Multiple Imputations by Chained Equation

We used the following variables to perform multiple imputation by chained equation in Stata

- Outcomes (broader sickness absence, alcohol-related sickness absence)
- covariates (sex, age group, household income, occupation, highest qualification, SEIFA, marital status, daily smoking)
- exposures (daily alcohol consumption and HED frequency.

We performed multiple imputations by chained equation (MICE) to account for missing data from our dataset using the 'mi impute' suites in Stata based on Rubin's rules (Alpman, 2016; Royston, 2014; Royston & White, 2011). The chained equation executed 6 imputations (the maximum of the universally accepted number of imputations 2-10)(Von Hippel, 2020). (Eddings & Marchenko, 2012).

```
Multivariate imputation                    Imputations =          6
Chained equations                                added =          6
Imputed: m=1 through m=6                       updated =          0

Initialization: monotone                    Iterations =         60
                                               burn-in =         10
```

any_absence: ordered logistic regression
any_ab~e_in_il: ordered logistic regression
totvol3_recode: ordered logistic regression
agegroup1470~e: ordered logistic regression
marstat: ordered logistic regression
comorbidity: ordered logistic regression
dailysmoking: logistic regression
recode_occup~n: ordered logistic regression
sex: logistic regression
recode_hhinc~e: ordered logistic regression
recode_educa~n: ordered logistic regression
seifaquintil~e: ordered logistic regression
asgs3: ordered logistic regression

	Observations per *m*			
Variable	Complete	Incomplete	Imputed	Total
any_absence	8118	1445	1445	9563
any_ab~e_in_il	7052	2511	2511	9563
totvol3_recode	9110	453	453	9563
agegroup1470~e	9563	0	0	9563
marstat	9527	36	36	9563
comorbidity	9481	82	82	9563
dailysmoking	9563	0	0	9563
recode_occup~n	9164	399	399	9563
sex	9563	0	0	9563
recode_hhinc~e	8447	1116	1116	9563
recode_educa~n	7458	2105	2105	9563
seifaquintil~e	9563	0	0	9563
asgs3	5368	4195	4195	9563

(Complete + Incomplete = Total; Imputed is the minimum across *m* of the number of filled-in observations.)

Sensitivity analysis for using complete case analysis (non-imputed dataset)

	**Alcohol-related absenteeism**								**Broader sickness absenteeism**							
	Unadjusted				Adjusted*				Unadjusted				Adjusted*			
	OR	95% CI		P	OR	95% CI		P	OR	95% CI		P	OR	95% CI		P
**Average daily drinking**																
Abstainers	-				-				0.76	0.62	0.93	**0.008**	**0.75**	**0.61**	**0.93**	**0.01**
Light to Moderate	Ref				Ref				Ref				Ref			
Risky	**3.70**	**2.24**	**6.12**	**<0.0001**	**3.64**	**2.15**	**6.16**	**<0.0001**	**0.81**	**0.63**	**1.04**	0.104	**0.84**	**0.65**	**1.10**	0.20
High Risk	**6.54**	**3.87**	**11.05**	**<0.0001**	**5.78**	**3.29**	**10.16**	**<0.0001**	1.00	0.74	1.36	0.985	**1.00**	**0.73**	**1.36**	0.98
**Heavy episodic drinking (HED)**																
Abstainers	-				-				0.91	0.69	1.20	0.502	0.90	0.68	1.18	0.44
Never	Ref				Ref				Ref				Ref			
Less than monthly	2.18	0.98	4.86	0.06	1.84	**0.80**	**4.21**	0.15	**1.39**	**1.16**	**1.65**	**<0.0001**	**1.30**	**1.08**	**1.56**	**0.01**
Monthly but less than weekly	3.34	1.63	6.87	**<0.0001**	**3.08**	**1.28**	**5.76**	**<0.0001**	**1.28**	**1.07**	**1.55**	**0.009**	1.14	0.93	1.38	0.21
Weekly or more	12.12	6.34	23.16	**<0.0001**	**11.68**	**4.72**	**19.24**	**<0.0001**	**1.18**	**0.97**	**1.44**	**0.047**	1.11	0.90	1.37	0.34

Adjusted for sex, age group, household income, occupation, highest qualification, SEIFA, marital status, daily smoking

Alpman, A. (2016). Implementing Rubin's alternative multiple-imputation method for statistical matching in Stata. *The Stata Journal*, *16*(3), 717-739.

Eddings, W., & Marchenko, Y. (2012). Diagnostics for multiple imputation in Stata. *The Stata Journal*, *12*(3), 353-367.

Royston, P. (2014). ICE: Stata module for multiple imputation of missing values.

Royston, P., & White, I. R. (2011). Multiple imputation by chained equations (MICE): implementation in Stata. *Journal of statistical software*, *45*, 1-20.

Von Hippel, P. T. (2020). How many imputations do you need? A two-stage calculation using a quadratic rule. *Sociological Methods & Research*, *49*(3), 699-718.
